# Health care providers' attitudes and counseling behaviors related to obesity

**DOI:** 10.1002/osp4.686

**Published:** 2023-05-25

**Authors:** Marjanna Smith, Christine Gallagher, Deanne Weber, William H. Dietz

**Affiliations:** ^1^ Milken Institute School of Public Health The George Washington University Washington District of Columbia USA; ^2^ Porter Novelli Public Services Washington District of Columbia USA

**Keywords:** obesity, obesity treatment, pharmacotherapy, provider attitudes

## Abstract

**Background:**

Obesity affects over 42% of the U.S. adult population, yet it remains undertreated. Many healthcare providers are biased in their perceptions and attitudes regarding obesity management and lack knowledge about how to treat it.

**Methods:**

The authors analyzed the results of the 2021 DocStyles survey to examine primary care providers' treatment and perceptions of obesity. The sample consisted of primary care physicians and nurse practitioners/physician assistants. Questions assessed healthcare providers' attitudes and counseling behaviors related to obesity, including referrals, use of medical therapy, barriers to care, and perceived risk factors for obesity.

**Results:**

1168 primary care providers who treat obesity participated in the survey. About half of the providers reported referring patients for obesity treatment. Almost two‐thirds of providers had prescribed anti‐obesity medications in the last 12 months. Those who did not prescribe anti‐obesity medications reported a lack of familiarity with the medications or concerns about safety. Over three‐quarters of providers indicated at least one barrier to treating obesity. Over half of the providers reported that poverty and food insecurity contributed significantly to the high prevalence of obesity in communities of color.

**Conclusion:**

Increased familiarity with anti‐obesity medications may improve treatment. Reasons for patients' low priority accorded to obesity care remain the focus of future research.

## 1 INTRODUCTION

Obesity affects over 42% of the U.S. adult population,^1^ yet it remains undertreated.^2^ Obesity is associated with increased risks of comorbidities including cardiovascular disease, diabetes, and cancer and early mortality.^3^ Obesity is also associated with increased healthcare costs and costs associated with lost productivity.^4^ The authors' previous investigations found that many healthcare providers have biased perceptions and attitudes regarding obesity management, or lack training to treat the disease.^5^ Insufficient treatment of obesity exacerbates its adverse consequences at both the individual and population level.

Previous work analyzing healthcare professionals' knowledge of obesity treatment has demonstrated a lack of familiarity with the clinical guidelines for obesity management.^2^, ^5^, ^6^ A previous survey of internists, family practitioners, obstetricians/gynecologists, and nurse practitioners conducted 5 years ago showed that only 16% of respondents indicated familiarity with the guidelines established by the U.S. Preventive Services Task Force and Centers for Medicare and Medicaid.^6^ Only 15% of respondents identified the correct indication to prescribe pharmacotherapy to treat obesity, and 20% of respondents indicated that long‐term pharmacotherapy is unsafe.^6^ Healthcare professionals have cited time constraints, limited reimbursement, higher priority concerns, and lack of training in obesity management as the main barriers that prevent them from effectively treating their patients.^2^, ^5^

Only limited research has focused on the rates of the use of pharmacotherapy to treat obesity. One large study of anti‐obesity medication (AOM) use in 2.2 million adults across eight large healthcare organizations found that only 1.3% of eligible patients received a prescription for an AOM over a period of 6 years.^7^ Another study of over 600 healthcare providers indicated that only 11% of providers recommended prescription weight loss medications to their patients as a treatment option.^2^ Research also indicates that rates of pharmacotherapy use appear to be increasing among primary care providers, while pharmacotherapy use among OB‐GYNs and nurse practitioners are much lower. The approval of multiple new medications for obesity treatment since 2012 is likely a catalyst for the observed increase in pharmacotherapy use among primary care providers.^8^

This study contributes to the literature by assessing the current practices and beliefs of primary care providers and evaluates practices and beliefs among primary care physicians (PCPs) and nurse practitioners/physician assistants (NP/PAs) regarding obesity counselling. This study reflects an ongoing inquiry into what primary care providers know about obesity care and identifies gaps in medical training and practice.

## 2 METHODS

The Spring DocStyles 2021 survey instrument was developed by Porter Novelli with technical guidance provided by federal public health agencies and other non‐profit and for‐profit clients. DocStyles contained 102 questions, some with multiple subparts, which were designed to provide insight into health care providers' attitudes and counseling behaviors in regard to a variety of health issues and to assess their use of available health information sources. Demographic data were collected on each respondent's gender, race, work setting, practice location, and years of practice. Respondents were asked if they referred patients with obesity, and if so, to what types of specialists or services. Respondents also indicated whether they had prescribed medication to treat obesity in the past year, and if so, which medications. Respondents who had not prescribed medication were asked for their reasons for not prescribing. Respondents also reported their own barriers to treating obesity as well as the barriers they perceived among their patients to receive obesity treatment. Respondents were also asked whether the COVID‐19 pandemic influenced the rigor of their treatment and how the use of telemedicine to treat obesity impacted their treatment. Lastly, respondents were asked for their perspectives on how social determinants of health such as race and food insecurity may influence the prevalence of obesity in communities of color. The survey was conducted by SERMO, a global market research company. Efforts were made to enroll 1000 PCPs (a mix of family practitioners and internists), 250 OB/GYNs, 250 pediatricians, and 250 NP/PAs. Respondents were paid an honorarium for completing the survey.

A total of 1002 PCPs and 251 NP/PAs participated in the survey. The response rate was 65% among PCPs and 71% among NP/PAs. The sample was a convenience sample and no information was available to compare respondents to non‐respondents. Respondents were screened to include only those who practice in the United States, actively see patients, work in an individual, group, or hospital practice, and had been practicing for at least 3 years. Respondents were not required to participate and could exit the survey at any time. To protect respondent confidentiality, no individual identifiers were included in the database. Respondents who reported that they did not treat obesity (*n* = 85) were excluded from the analysis, leaving an analytic sample of 954 PCPs and 214 NP/PAs. Results from pediatricians in this report were excluded because concerns and practices related to the prevention and treatment of childhood obesity differ significantly from adults.

Demographic information for PCPs and NP/PAs is provided in Table 1. The average age of participants was 47 years (*M* = 46.8, SD = 11.7); PCPs were slightly older, and the average length of practice was 16 years (*M* = 16.4, SD = 9.7). The majority of respondents were White, with NP/PAs having the largest percentage of White respondents. Men made up 61% of the sample and were more likely to be PCPs. Women made up the majority of the NP/PA respondents. Most providers worked in a group outpatient practice or clinic and a majority worked in a suburban setting.

**TABLE 1:** Characteristics of survey respondents.

	Total	PCPs	NP/PAs
*n*	1168	954	214
Age (mean)	46.8	47.7	42.9
Gender			
Male	61%	69%	25%
Female	39%	31%	75%
Race			
White	69%	65%	88%
Asian	22%	25%	8%
Other race	3%	4%	1%
Black or African American	3%	3%	1%
Two or more races	3%	3%	1%
Native Hawaiian or other Pacific Islander	<1%	<1%	<1%
American Indian or Alaska Native	<1%	<1%	<1%
Work setting			
Group outpatient practice or clinic	64%	64%	64%
Individual outpatient practice	18%	17%	20%
Inpatient practice/hospital	18%	18%	17%
Practice location			
Urban	36%	36%	36%
Suburban	53%	54%	49%
Rural	12%	11%	15%
Years of practice (mean)	16.4	17.0	13.9

## 3 RESULTS

### 3.1 Referral practices

The referral practices for obesity across provider types are shown in Table 2. Slightly over half of the total primary care providers surveyed reported that they referred patients for obesity treatment, while four out of ten said they provided counseling and treatment in their office. PCPs were more likely than NP/PAs to indicate that they provided in‐office counseling and treatment. Of the primary care providers who referred patients with obesity, most referred to registered dieticians and obesity specialists; more than a third referred to community‐based and face‐to‐face weight loss programs. There were no statistically significant differences in where PCPs and NP/PAs referred patients with obesity.

**TABLE 2:** Referrals for obesity across providers.

	Total	PCPs	NP/PAs
	*N* = 1168	*N* = 954	*N* = 214
Do you refer patients with obesity?			
Yes, I refer patients	55%	54%	60%
No. I do not have anywhere to refer patients	5%	4%	8%
No. I provide counseling and treatment in the office*	40%	41%	32%
Where do you refer patients with obesity?^a^			
Registered dietician or nutritionist	79%	78%	86%
Obesity specialist	67%	68%	61%
Community‐based weight loss program	39%	39%	39%
Face‐to‐face weight loss program	39%	39%	36%
Digital weight loss program	20%	21%	18%
Somewhere else not listed	8%	7%	11%

^a^ Multiple selections.

*p value <0.05 in difference in response between providers.

### 3.2 Medical therapy practices

Medical therapy practices across providers are shown in Table 3. Almost two‐thirds of primary care providers indicated that they had prescribed a medication for obesity in the last 12 months. The most frequently selected drugs were Phentermine HCl, Contrave, and Saxenda. PCPs were more likely than NP/PAs to have prescribed medication overall, as well as each of the listed medications. While approximately one‐third of providers indicated that they had not prescribed a medication for obesity in the last 12 months, nearly one‐third of providers indicated that they had prescribed three or more medications in the last 12 months. The most frequently cited reasons for not prescribing medications for obesity were lack of familiarity with AOMs and concerns about drug safety. A similar percentage of PCPs and NP/PAs said they lacked familiarity with obesity medications. PCPs were almost two times more likely than NP/PAs to indicate concerns about drug safety and 21% of PCPs indicated that they thought medical therapy was not effective.

**TABLE 3:** Medical therapy for obesity across providers.

	Total	PCPs	NP/PAs
	*N* = 1168	*N* = 954	*N* = 214
Which of these medications have you prescribed for obesity in the last 12 months?^a^			
Phentermine HCl*	38%	41%	26%
Buproprion HCl/naltrexone HCl (Contrave)*	37%	40%	22%
Liraglutide (Saxenda)*	34%	38%	16%
Orlistat (Xenical)*	30%	33%	12%
Phentermine HCl/topiramate ER (Qsymia)*	27%	30%	15%
I haven't prescribed medication for obesity*	35%	31%	56%
How many medications have you prescribed in the last 12 months?			
None*	35%	31%	56%
One	17%	17%	16%
Two or more*	48%	53%	28%

	Total *N* = 413	PCP *N* = 294	NP/PAs *N* = 119
Why have you not prescribed medication for obesity in the last 12 months?^b^			
Lack of familiarity	44%	44%	45%
Concern about drug safety*	41%	47%	26%
Medical therapy is not effective*	17%	21%	8%
Obesity does not require medical therapy	3%	4%	1%
Obesity is not a disease	<1%	1%	0%
Other reasons not listed*	37%	31%	52%

^a^ Multiple selections.

^b^ Percent among those who have not prescribed medication; multiple selections.

*p value <0.05 in difference in response between providers.

### 3.3 Barriers to obesity treatment

Table 4 describes the respondents' perceived barriers to obesity treatment. Over three‐quarters of the primary care providers surveyed indicated at least one barrier to treating obesity. The top three reported barriers were that their patients had other higher priority issues, they were not adequately compensated for treating obesity, or they did not have time to treat obesity. NP/PAs were less likely than PCPs to indicate that they are not adequately compensated for treating obesity. In addition to these barriers, 13% of primary care providers indicated that obesity is caused by patients themselves. NP/PAs were less likely than PCPs to select this response. Only two percent of providers indicated that obesity is not a disease.

**TABLE 4:** Barriers to obesity treatment.

	Total	PCPs	NP/PAs
	*N* = 1168	*N* = 954	*N* = 214
My barriers to treating obesity include			
Patients have other higher priority issues	51%	51%	51%
I am not adequately compensated for it*	20%	22%	12%
I do not have time to treat obesity	19%	19%	19%
I am not up‐to‐date on obesity treatments	18%	17%	22%
Obesity is caused by patients themselves*	13%	15%	7%
Obesity is not a disease	2%	2%	2%
None of the above	24%	24%	26%
What barriers do your patients encounter when addressing their obesity?^a^			
Inadequate insurance coverage for obesity care	61%	61%	61%
Affordability of commercial/digital weight loss programs	57%	56%	60%
Lack of recognition that obesity is a chronic disease	49%	48%	51%
Lack of access to specialty care for obesity	40%	39%	44%
Availability of grocery stores with fresh, healthful food	37%	37%	41%
Availability of safe places to exercise	29%	29%	28%
Accessibility of commercial/digital weight loss programs	31%	30%	38%
None of the above	5%	6%	3%

^a^ Multiple selections.

*p value <0.05 in difference in response between providers.

When asked about barriers that their patients encountered when addressing their obesity, primary care providers were most likely to select inadequate insurance coverage and affordability of weight loss programs. Almost half of the providers indicated that the lack of recognition that obesity is a chronic disease was a patient barrier. There were no statistically significant differences between PCPs and NP/PAs regarding this question.

### 3.4 Effects of COVID‐19 on obesity treatment

The survey included questions on the impact of the COVID‐19 pandemic on obesity treatment because several preliminary reports indicated that the pandemic resulted in an increase in the prevalence of obesity in children and adults,^9^, ^10^ although a larger study of adults failed to document an increase.^11^ The majority of primary care providers said that their treatment level was about the same now compared to before the COVID‐19 pandemic (72%); 20% were treating more aggressively and 8% were treating less aggressively. The survey also asked respondents about their use of telemedicine to treat obesity because the pandemic contributed to an expansion of telemedicine services, and research suggests that patients who received obesity care via telemedicine during the pandemic experienced higher odds of weight loss compared to those who did not.^12^ Seventeen percent of primary care providers indicated that telemedicine made obesity care easier and 38% stated that it had no impact (38%); 27% of PCPs said it made obesity care more difficult. PCPs were slightly more likely than NP/PAs to use telemedicine to treat obesity (83% vs. 75%) and more likely to say that telemedicine did not impact obesity care (40% vs. 29%).

### 3.5 Communities of color

Another key issue related to obesity is racial disparities in the prevalence of food insecurity in the United States, as food insecurity disproportionately affects racial and ethnic minority groups^13^ and is significantly associated with increased adiposity.^14^ In 2018, households headed by Black or Hispanic parents experienced food security at a rate of 21% and 16%, respectively, compared to the national average of 11%.^15^ The racial and ethnic disparities observed with food insecurity have only been exacerbated by the COVID‐19 pandemic. In 2020, approximately 25% of families with school‐age children reported food insecurity in the last 30 days; this rate was 40% among families in which the parents were either Black or Hispanic.^16^ Eighty‐five percent of providers thought that poverty and food insecurity contributed “somewhat” or “a lot” to the high prevalence of obesity in communities of color, while only 50% of providers thought that stress caused by racism or discrimination contributed “somewhat” or “a lot” to the issue. Many providers also thought that in communities of color, lack of awareness of obesity as a disease (77%) and cultural norms that accept increased body size (80%) also contributed “somewhat” or “a lot” to high obesity prevalence. No statistically significant differences were found between PCPs and NP/PAs in their views on contributors to high obesity prevalence in communities of color.

### 3.6 Perspective on obesity etiology

Along with individually reviewing the results of the survey question “My barriers to treating obesity include…”, the authors explored whether a provider's response to this question was related to their responses to other survey questions. Of particular interest was the response option, “Obesity is caused by patients themselves.” Thirteen percent of participants selected this response. Primary care physicians who thought that patients with obesity were responsible for their obesity were more likely to think that obesity was not a disease (8% vs. 1%). When asked about reasons for not prescribing medication for obesity, participants who reported that obesity was caused by patients themselves were more likely to select “concern about drug safety” (60% vs. 39%) and “obesity doesn't require medical therapy” (15% vs. 2%). They were also more likely to select “Medical therapy is not effective” (30% vs. 16%). In terms of perceived patient barriers, participants who felt that obesity was caused by patients themselves were more likely to report that the lack of recognition that obesity is a chronic disease is a barrier that patients encounter (59% vs. 47%).

### 3.7 Prescribers versus non‐prescribers

Comparisons of the referral practices, provider barriers to treatment, and perceived patient barriers to treatment among prescribers versus non‐prescribers of AOMs are shown in Table 5. Providers who had prescribed AOMs in the last 12 months were more likely to provide obesity counseling and treatment in the office, compared to those who had not prescribed AOMs in the last 12 months. Prescribers were also more likely to refer patients with obesity to face‐to‐face weight loss programs and digital weight loss programs compared to non‐prescribers. Prescribers were more likely to indicate that inadequate insurance coverage and affordability of commercial/digital weight loss programs were barriers that their patients encountered when addressing their obesity.

**TABLE 5:** Responses stratified by prescribers and non‐prescribers of obesity medication.

	Prescribers (*N* = 755)	Non‐prescribers (*N* = 413)
Do you refer patients with obesity?		
Yes, I refer patients.*	52%	62%
No. I provide counseling and treatment in the office.*	46%	28%
No. I do not have anywhere to refer patients.*	3%	10%
Where do you refer patients with obesity?^a^		
Registered dietician	79%	79%
Obesity specialist*	70%	62%
Face‐to‐face weight loss program*	43%	32%
Community‐based weight loss program	41%	36%
Digital weight loss program*	24%	15%
Somewhere else not listed	7%	9%
My barriers to treating obesity include:		
Patients have other higher priority issues	50%	53%
None of the above*	27%	19%
I am not adequately compensated for it	22%	17%
I do not have time to treat obesity*	16%	24%
Obesity is caused by patients themselves*	16%	10%
I am not up‐to‐date on obesity treatments*	14%	25%
Obesity is not a disease*	3%	<1%
What barriers do your patients encounter when addressing their obesity?		
Inadequate insurance coverage*	66%	51%
Affordability of commercial/digital weight loss programs*	60%	51%
Lack of recognition that obesity is a chronic disease	47%	52%
Lack of access to specialty care for obesity	39%	43%
Availability of grocery stores with fresh, healthful food	37%	38%
Accessibility of commercial/digital weight loss programs	32%	29%
Availability of safe places to exercise	30%	27%
None of the above*	4%	7%

^a^ Multiple selections.

*p value <0.05 in difference in response between prescribers versus non‐prescribers.

Providers who had not prescribed AOMs in the last 12 months were more likely to select “I do not have anywhere to refer patients” than prescribers. Non‐prescribers were also more likely to indicate that they did not have time to treat obesity and were not up to date on obesity treatments.

## 4 DISCUSSION

This analysis of the results of the 2021 DocStyles survey illuminated multiple key findings. The differences in treatment practices between PCPs and NP/PAs may reflect differences in training and competencies by provider type. Because obesity affects over 42% of U.S. adults, understanding the attitudes and barriers related to treatment is a critical concern. Furthermore, understanding the attitude and practices of the frontline clinicians who encounter patients with obesity daily will help to target the needs for further education and training.

First, the analysis of referral practices of the survey respondents revealed that of those who treat obesity, over half of the providers refer patients with obesity. Of the providers who refer patients, the majority refer patients to registered dieticians or nutritionists as well as obesity specialists. When asked about their own barriers to treating obesity, the most common answer was “patients have other higher priority issues”; approximately half of the providers selected this response. The most frequently cited patient barriers were “Inadequate insurance coverage for obesity care” and “Affordability of commercial/digital weight loss programs,” and about half of the respondents responded that “Lack of recognition that obesity is a chronic disease” as a patient barrier.

Comparison of providers who had prescribed AOMs versus those who had not revealed key findings on obesity treatment practices and attitudes among primary care providers. Prescribers of AOMs were significantly more likely to provide treatment and counseling in their office, while non‐prescribers were significantly more likely to refer patients for obesity treatment. Non‐prescribers were also more likely than prescribers to indicate that they did not have anywhere to refer patients, they lacked the time to treat obesity, were not up to date on obesity treatments, and indicated concerns and lack of knowledge about drug safety. All these findings reflect the need for increased awareness of obesity treatment resources and training.

The findings that almost 50% of providers lack familiarity with drug therapy, and almost as many PCPs are concerned about drug safety, present significant challenges to the adequacy of care. The FDA approval of semaglutide and the pending approval of tirzepatide offer new and highly effective options with minimal side effects for the treatment of obesity. For example, 35% of patients in a semaglutide trial and 63% of patients in a tirzepatide (15 mg dose) trial lost ≥20% of their body weight.^17^, ^18^ These findings emphasize the need for inclusion of obesity in medical school curricula, and training in the use of AOMs for interns, residents, and practicing PCPs. The lack of patient coverage for obesity as well as the lack of reimbursement for obesity care represent additional barriers to the delivery of care.^19^

Sixty‐five percent of the sample prescribed medication for obesity. A limitation of this finding is that this statistic does not indicate the extent to which these prescriptions are filled by patients. According to the findings of a large study of AOM use, only 1.3% of patients with obesity filled an AOM prescription, and only a small proportion of prescribers were responsible for the majority of filled prescriptions.^7^ This observation suggests a gap in the treatment process and frequent failure to get patients to follow‐through with their prescribed treatment. The high rate of respondents stating that they prescribed pharmacotherapy may also suggest that this sample is biased compared to the general population of providers. In future studies, data on the number of providers prescribing AOMs and the rate of filled prescriptions per provider would provide essential insight into patient adherence as a function of providers' quality of obesity counselling and explanation of drug therapies to their patients.

The majority of primary care providers reported that their treatment level was about the same now compared to before the COVID‐19 pandemic. A possible explanation for this result is the lack of significant changes in obesity prevalence among adults as a result of the COVID‐19 pandemic.^11^ Preliminary findings on the impact of the COVID‐19 pandemic on obesity treatment indicate that behavioral weight‐management interventions do not differ significantly in effectiveness when delivered using telemedicine technology rather than in person.^20^ This observation supports the utility and effectiveness of telemedicine to treat obesity and should be considered by healthcare providers in their efforts to improve the quality and accessibility of obesity treatments for their patients.

Regarding social determinants of health, most respondents reported that poverty and food insecurity contributed to the high prevalence of obesity in communities of color, indicating an awareness of these as social determinants of obesity. Despite these findings, only half of the respondents thought that stress caused by racism or discrimination contributed to the high prevalence of obesity in communities of color, emphasizing the need for improved awareness and understanding of racism as a risk factor. This finding indicates a need for greater focus on social determinants of health among providers as well as an opportunity to mobilize providers as advocates for social change.

In conclusion, improved familiarity with obesity treatment methods, particularly AOMs, may greatly impact treatment. Future research on the prioritization of obesity care by providers and patients is needed to better understand this issue.

## CONFLICT OF INTEREST STATEMENT

The authors report funding from Novo Nordisk, WW International, Pfizer, Currax, Seca and Eli Lilly, outside the submitted work. In addition, Dr. Dietz reports consulting fees from the Roundtable on Obesity Solutions of the National Academy of Medicine, outside the submitted work.
